# An evaluation of contralateral hand involvement in the operation of the Delft Self-Grasping Hand, an adjustable passive prosthesis

**DOI:** 10.1371/journal.pone.0252870

**Published:** 2021-06-17

**Authors:** Alix Chadwell, Natalie Chinn, Laurence Kenney, Zoë J. Karthaus, Daniek Mos, Gerwin Smit

**Affiliations:** 1 Centre for Health Sciences Research, University of Salford, Salford, United Kingdom; 2 Department of Biomechanical Engineering, Delft University of Technology, Delft, The Netherlands; University of Electronic Science and Technology of China, CHINA

## Abstract

The Delft Self-Grasping Hand is an adjustable passive prosthesis operated using the concept of tenodesis (where opening and closing of the hand is mechanically linked to the flexion and extension of the wrist). As a purely mechanical device that does not require harnessing, the Self-Grasping Hand offers a promising alternative to current prostheses. However, the contralateral hand is almost always required to operate the mechanism to release a grasp and is sometimes also used to help form the grasp; hence limiting the time it is available for other purposes. In this study we quantified the amount of time the contralateral hand was occupied with operating the Self-Grasping Hand, classified as either direct or indirect interaction, and investigated how these periods changed with practice. We studied 10 anatomically intact participants learning to use the Self-Grasping Hand fitted to a prosthesis simulator. The learning process involved 10 repeats of a feasible subset of the tasks in the Southampton Hand Assessment Procedure (SHAP). Video footage was analysed, and the time that the contralateral hand was engaged in grasping or releasing was calculated. Functionality scores increased for all participants, plateauing at an Index of Functionality of 33.5 after 5 SHAP attempts. Contralateral hand involvement reduced significantly from 6.47 (first 3 attempts) to 4.68 seconds (last three attempts), but as a proportion of total task time remained relatively steady (increasing from 29% to 32%). For 9/10 participants most of this time was supporting the initiation of grasps rather than releases. The reliance on direct or indirect interactions between the contralateral hand and the prosthesis varied between participants but appeared to remain relatively unchanged with practice. Future studies should consider evaluating the impact of reliance on the contralateral limb in day-to-day life and development of suitable training methods.

## Background

Upper-limb prosthetic hands can be categorised as active or passive. With active prostheses the user has continuous control over the hand posture, opening and closing the hand either through movements of the body (e.g. via a shoulder-worn harness), or via electric motors controlled from electromyographic signals. In contrast, passive hands offer limited or no grasping function, but have traditionally been favoured for their cosmetically appealing appearance, their light weight, and their low complexity. According to literature, about one third of users of hand prostheses, use passive devices [1]. Passive prostheses can be categorised into either static or adjustable designs. Adjustable passive hands typically need support from the contralateral limb to perform a grasp [1].

**Fig 1:**
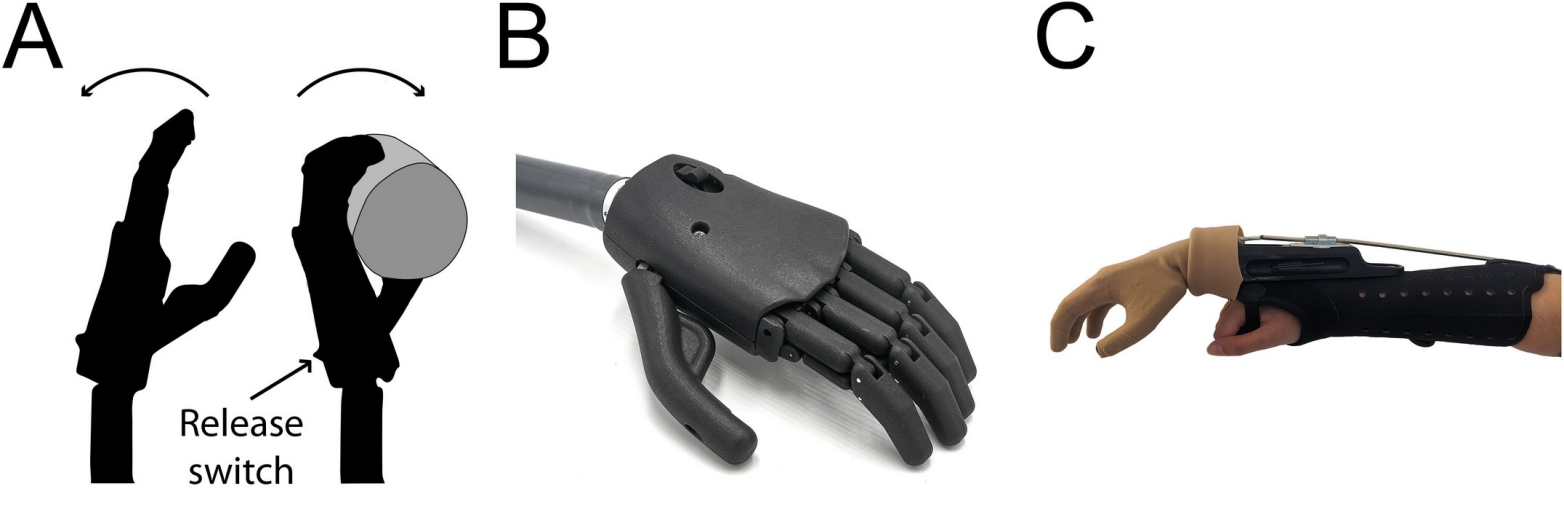
The Self-Grasping Hand. A: As the Self-Grasping Hand is flexed backward at the wrist, the fingers curl around the object. **B:** Self-Grasping Hand prototype. **C:** Self-Grasping Hand attached to the TRS simulator forearm brace with a cosmetic glove worn over the hand (Please note that the TRS simulator is designed for use with body-powered prostheses and therefore includes a cable running along the brace. This cable was not attached to the Self-Grasping Hand and was not used during this study).

One such device is the ‘Self-Grasping Hand’ developed at the Delft University of Technology (TU Delft) [2, 3]. The hand is operated using the concept of tenodesis (where opening and closing of the hand is mechanically linked to the flexion and extension of the wrist). As the prosthetic wrist is extended, a linkage mechanism causes the fingers of the hand to curl closed (**Fig 1A**), and a ratchet is engaged in a discrete number of locations to maintain a locked grasp. This wrist extension can be achieved by pushing against the palm of the hand or the thumb. To open the hand, the user must engage a switch on the rear of the palm (**Fig 1A**) and provide a small amount of additional wrist extension to disengage the ratchet. The hand will then open all the way to its resting posture. The thumb can be abducted radially into a position flat to the palm (as shown in **Fig 1B**), or rotated round into an opposition position against the index finger (more similar to the position in **Fig 1A–1C**), or any point in-between.

Adjustable passive hands can be of particular benefit to users who would like a lightweight, functional hand. Adjustable passive hands are purely mechanical, but unlike active devices do not rely on a restrictive harness system for their control [4]. However, unlike an active prosthesis, the Self-Grasping Hand always requires some involvement from either the contralateral hand or the environment during both grasp and release. Use of the contralateral hand reduces the time during which it is available to engage in other parts of the task or in alternative tasks. We know that for people with unilateral limb absence the contralateral hand often plays a role in the grasping and manipulation of objects [5–7], with users stabilising objects, placing objects into their prosthetic hand, or manually adjusting prosthetic wrist/thumb positions. Nevertheless, the extent of contralateral hand involvement in tasks, and whether this reduces with practice, or can be reduced through specific training regimes has yet to be explored. The video-based methodology introduced here, to identify types of contralateral hand involvement during use of the Self-Grasping Hand, could therefore be of value in studies of other prosthetic hands.

In this study, we aim to investigate the time the contralateral hand is occupied during task performance and changes to this as a person learns to use the Self-Grasping Hand. We hypothesise that, with practice, participants will become faster at using the Self-Grasping Hand, and that the time during which the contralateral hand is engaged in grasping/releasing will reduce. As the contralateral hand is almost always required to operate the releasing mechanism, but is not always required for grasping, we also hypothesise that the time during which the contralateral hand is engaged in grasping will be lower than the time spent supporting release.

When using another type of prosthesis, grasping/releasing would normally take one of two forms: (1) the hand would grasp/release an object (termed direct grasping), or (2) an object would be placed into the hand by the other hand (termed indirect grasping). In the case of the Self-Grasping Hand, there is also a third option: (3) the contralateral hand can press on the fingers of the prosthetic hand to close it around an object, or press the button on the dorsal surface of the hand to open it. In this manuscript we are focussed on the interaction of the contralateral hand with the prosthesis. Therefore, when we refer to a ‘direct interaction’, this refers to option 3 above, where the contralateral hand makes direct contact with the prosthesis; when we refer to an ‘indirect interaction’ we refer to option 2 above, where the contralateral hand is indirectly interacting with the prosthesis via another object.

In this manuscript we present a video-based coding structure which can be used to quantify contralateral hand involvement when performing tasks using a prosthesis. This is the first study aiming to describe the patterns of use of the contralateral hand and changes to this with practice. It is also the first study to quantify and highlight observations relating to the functionality provided by the Self-Grasping Hand.

## Methods

### Participants

A convenience sample of 10 right-handed anatomically intact participants with no upper-limb impairments (3 male/7 female) were recruited from the students at the University of Salford. Ethical approval for the study was granted by the University of Salford Health Research Ethics committee (REF: HSR1819-087) and informed consent was gained from all participants.

### The prosthesis

A right-hand prototype of the Self-Grasping Hand was provided by TU Delft (**Fig 1B**). Pilot work showed that use of a cosmetic glove improved conformability of the hand around the object and grasp security, due to the compliance and friction of the glove material. For the first 3 participants, the hand was covered with a Steeper size L6 PVC glove (CGL6/E4), and for participants 4 to 10, a Steeper silicone glove (SGL6/E4) was worn. The change of glove coincided with replacement of the hand due to mechanism failure. The hand was connected to a TRS body-powered prosthesis simulator (TRS Inc, Boulder, Colorado, USA) consisting of a forearm/wrist brace weighing ~500g. The wrist of the simulator was not adjustable. The simulator allows use of the Self-Grasping Hand by anatomically intact participants as an extension of their own arm (**Fig 1C**). Although this simulator included a harness-driven cable system, this was disengaged for the purposes of this work.

### Protocol

#### Overview of the Southampton Hand Assessment Procedure (SHAP)

To record the change in function with practice, the Southampton Hand Assessment Procedure (SHAP) was used. Full details are available at http://www.shap.ecs.soton.ac.uk/. The SHAP includes 26 table-top tasks where participants are required to manipulate light and heavy abstract objects and undertake Activities of Daily Living (ADLs) such as pouring liquid from a carton. The time taken to complete each of the 26 tasks was recorded on a timer, activated by the participant according to the published SHAP protocol [8]. These times were then input into the online SHAP analysis software, which output an Index of Functionality (IoF) score normalised to 100 [9]. An IoF score of 100 would indicate normal hand function. IoF scores for prosthesis users are much more variable, most often falling between ~30–70 [10–13], but occasionally dropping below 10 [14] or exceeding 80 [15]. There are currently no studies presenting IoF scores for people using passive prosthetic hands, although data have been published for a passive finger prosthesis [16]. For the purposes of the assessing the Self-Grasping Hand, some tasks were excluded from the assessment (19/26 included). The reasons for this and the impacts of excluding tasks are detailed below.

#### Summary of protocol

**Fig 2:**
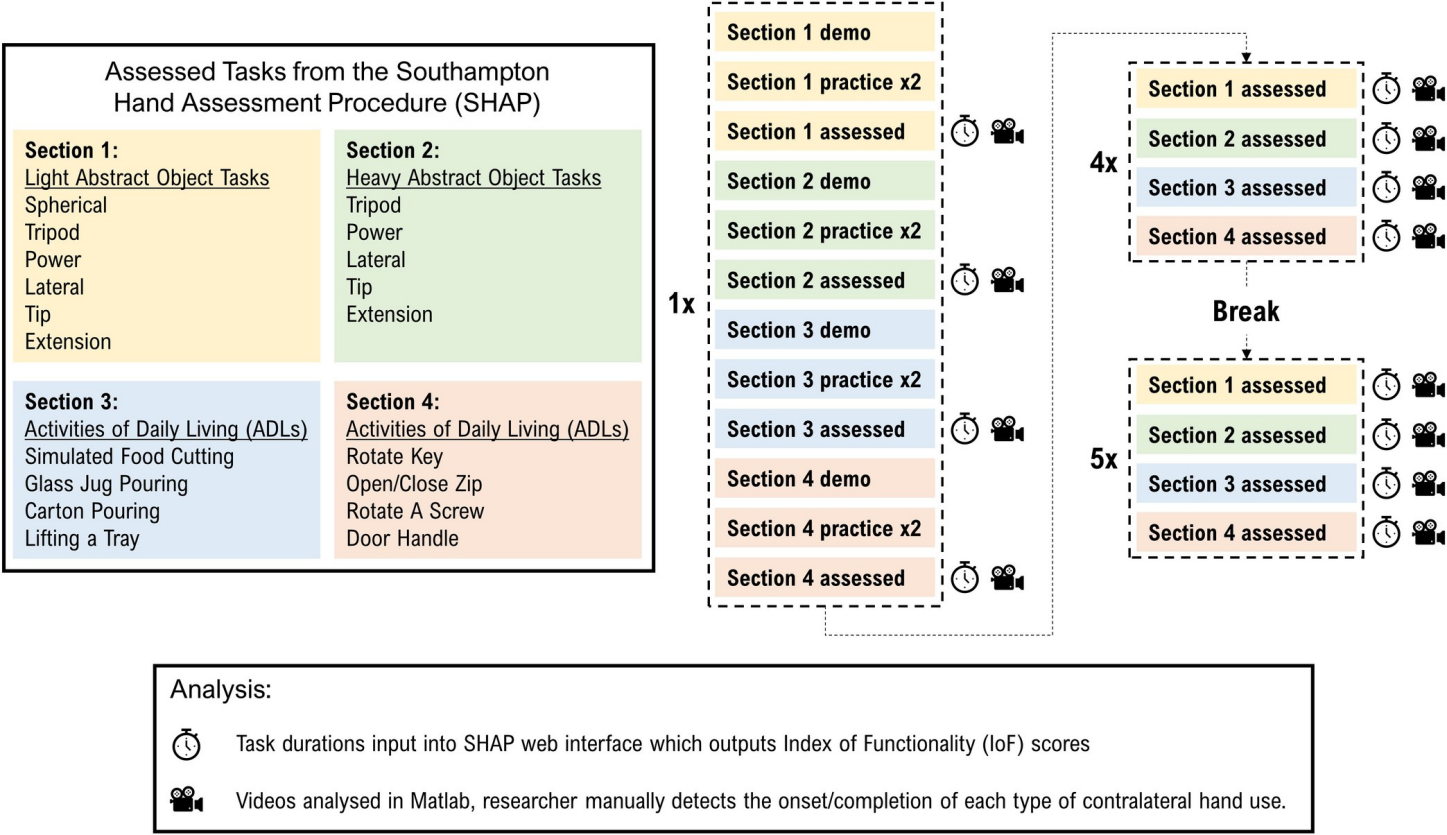
Protocol summary. **(Left)** The included tasks from the Southampton Hand Assessment Procedure (SHAP) were grouped into 4 sections. **(Right)** During the 1^st^ attempt, the researcher demonstrated the tasks in each section, the participant was then given 2 opportunities to practice before the timed and video recorded attempt. For all further attempts, no opportunities to practice were given prior to the assessed attempts. **(Bottom)** Analysis of the SHAP completion time was undertaken using the online SHAP analysis software. Analysis of the time spent using the contralateral hand was undertaken through manual review of the video data.

In order to assess how involvement of the contralateral hand changed as a person learned to use the Self-Grasping Hand, participants undertook the SHAP 10 times. A short break was given between attempts 5 and 6 and participants were able to request further breaks when needed. Each individual assessment (all 19 tasks) was completed before starting the next assessment. The data collection period lasted approximately 3–3.5 hours per participant. Task performance was recorded using a video camera to enable later analysis of contralateral hand use. To aid recording, the SHAP assessment was split into 4 sections: (1) the light abstract tasks, (2) the heavy abstract tasks, (3) the food cutting, glass jug pouring, carton pouring, and tray tasks, and (4) the key, zip, screwdriver, and handle tasks. During the first attempt at SHAP, at the start of each section, the researcher demonstrated all tasks in the section once. The SHAP website (http://www.shap.ecs.soton.ac.uk/) provides demonstration videos and clear guidelines for the researcher to follow. The participant was then given the opportunity to practice those tasks twice, before beginning the section under experimental conditions. No further practice trials were given for SHAP attempts 2–10. An overview of the experimental protocol is shown in **Fig 2**.

#### Changes made to the conventional SHAP assessment

Pilot work showed that the geometry of the Self-Grasping Hand meant that some of the SHAP tasks were unable to be completed (full details on the hand dimensions are provided in the S3 File). The tasks ‘Lifting a heavy object’ and ‘Unscrewing a jar lid’ both required the Self-Grasping Hand to hold a standard sized glass jar. ‘Lifting a light object’ required grasping of a standard sized food tin. The Self-Grasping Hand’s aperture (3.5cm) was too small to grasp the jar (~7cm) or tin (~7.5cm); therefore, it was not possible for participants to complete these three tasks. The diameter of the ‘Heavy spherical object’ (a metal sphere) sat between two of the discrete locking settings, and hence this stiff object was also not graspable. Although the ‘Light spherical object’ (a balsa wood sphere) was the same diameter as the ‘Heavy spherical object’, it was possible to hold this in place in the hand with friction despite the fingers not being able to tightly grasp it.

Pilot testing using a PVC glove showed that the ‘Pick-up-coins’, ‘Page-turning’ and ‘Button board’ tasks were unable to be performed with the hand, due to a combination of the discrete grasp apertures and the thumb positioning.

To reduce the assessment time, it was decided that these 7 tasks would not be assessed, and the maximum time of 100 seconds was allocated for these tasks. **Table 1** shows the tasks which were assessed.

**Table 1:** Tasks involved in the SHAP assessment.

**Abstract Objects**			
Light spherical	✓	Heavy spherical	✘
Light tripod	✓	Heavy tripod	✓
Light power	✓	Heavy power	✓
Light lateral	✓	Heavy lateral	✓
Light tip	✓	Heavy tip	✓
Light extension	✓	Heavy extension	✓
**Activities of Daily Living (ADLs)**			
Pick up coins	✘	Lifting a heavy object	✘
Button board	✘	Lifting a light object	✘
Simulated food cutting	✓	Lifting a tray	✓
Page turning	✘	Rotate a key	✓
Jar lid	✘	Open/close zip	✓
Glass jug pouring	✓	Rotate a screw	✓
Carton pouring	✓	Door handle	✓

Tasks marked with a cross were deemed not achievable within the time limit. These tasks were not assessed and were allocated the maximum time score to reduce the testing time.

The silicone glove offered more flexibility regarding the thumb positioning, however, as this change was made partway through the study, we did not assess whether any of the 7 excluded tasks were achievable with the change of glove.

It should be noted that the SHAP typically evaluates the performance of the assessed hand, without support from the contralateral hand in the completion of the tasks. For the Self-Grasping Hand, the SHAP tasks required contralateral hand involvement in the grasping and releasing of objects. Further, some of the grasps used did not match those identified in the SHAP guidelines. These factors were consistent across all participants and, as noted by Kyberd, this approach to completing SHAP does not impact on the validity of the assessment [17]. Further details on the grasps used are provided in the results section.

### Video data analysis

Code was written in Matlab (The Mathworks Inc, Cambridge, Cambridgeshire, England) to enable the researcher to view the videos and manually label frames according to contralateral hand use. Duration of contralateral hand use was separated into four categories (example videos of each type of grasp demonstrated by one of the co-authors are provided as S1 File, the individual in this manuscript has given written informed consent (as outlined in PLOS consent form) to publish these details):

1. Direct interaction during grasp—contralateral hand contacts the prosthesis to adjust the Self-Grasping Hand into a grasp
2. Direct interaction during release—contralateral hand contacts the prosthesis to activate the releasing mechanism
3. Indirect interaction during grasp—contralateral hand stabilises an object so that the Self-Grasping Hand can push the object into itself, or the contralateral hand pushes the object into the Self-Grasping Hand
4. Indirect interaction during release—contralateral hand removes an object from the Self-Grasping Hand, or uses an object to activate the releasing mechanism

The video data were labelled as follows:

For the direct interactions, the start was defined as the first frame which showed movement of the contralateral hand towards the prosthesis, providing contact with the prosthesis was subsequently made. The end time was defined as the first frame showing movement of the contralateral hand away from the prosthesis.

For the indirect interactions, the start was defined as the first frame which showed movement of the contralateral hand towards the object, providing the object was subsequently grasped. The end time was defined as the first frame showing movement of the contralateral hand away from the object.

If the contralateral hand grasped the SHAP case, plasticine (simulated sausage for ‘Food cutting task’), jar, tray (when not directly relating to the Self-Grasping Hand’s grasp or release action) or arrow mount (a component of the ‘Screwdriver task’) to support them this was not coded as it was counted as a necessary supporting action. Additionally, where the contralateral hand assisted with the location of the screwdriver in the screw head, this was not coded.

### Statistical analysis

#### Index of functionality

For each participant, the IoF for each attempt was calculated using the SHAP web interface and a summary box plot is presented. To determine whether there had been an improvement in function, for each participant the mean IoF for the 1^st^ and last 3 attempts was also calculated; these were compared using a paired sample t-test as described below. We hypothesised that the mean IoF score across the last 3 attempts would be higher than for the first 3 attempts.

#### Duration of contralateral hand involvement

Due to the time-consuming nature of the video analysis, only the first and last 3 attempts at SHAP were analysed and summary data excludes attempts 4–7.

The time spent using the contralateral hand was calculated for each individual task. When summarising the time in which the contralateral hand was involved in grasping or releasing, or when it was in direct or indirect contact with the prosthetic hand, we present the grand mean (±SD); this is calculated by calculating the mean for each participant across all tasks over all 10 attempts and then presenting the mean (±SD) of these mean values across all 10 participants.

Where we refer to changes in contralateral hand use over the assessment period, the mean time was calculated for each participant across the first or last 3 attempts, followed by the calculation of the grand mean across all 10 participants. To determine whether changes in performance were significant, we compared the grand mean of the first three attempts against the grand mean of the last three attempts using paired sample t-tests as described below.

We hypothesised that the mean contralateral hand use in seconds across the last 3 attempts would be shorter than for the first 3 attempts.

#### Paired samples t-test

To explore whether there were significant changes in performance, the means from all 10 participants were compared across 2 conditions using paired samples t-tests. The difference between the first and last attempts were deemed significant if p<0.05. T values are presented, and effect sizes are given using Cohen’s-d (mean difference/standard deviation). Outliers were labelled according to the outlier labelling rule 2.2*IQR [18]. The paired-samples t-test is robust to violations of normality with respect to Type I error [19–22]. therefore where assumptions of normality were violated, we state this, and continue with our analysis. We also present the z-values for skewness and kurtosis; z was deemed significant (p<0.05) if |z|>1.96 [23].

## Results

An excel spreadsheet is provided in the S2 File containing the breakdown of time spent completing each task and the time during which the contralateral hand was directly or indirectly involved in grasping or releasing. We highlight participants/tasks/attempts which were excluded from the video analysis and provide all the SHAP IOF scores.

### Index of functionality

**Fig 3:**
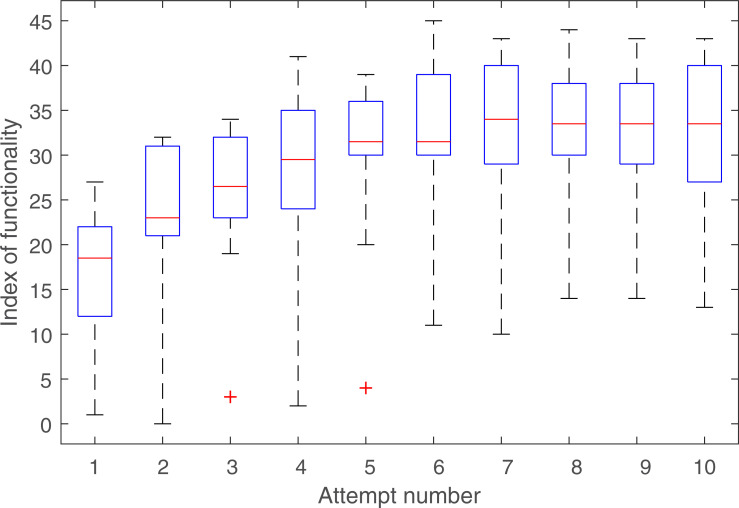
Box plot of SHAP IoF scores across all 10 participants. Outliers were labelled according to 2.2*IQR [18]. Participant 7 performed the tasks slower than the other participants explaining the long whiskers for the lower bound, however the improvement trend was the same, and there were no outliers when looking at the differences in SHAP score between attempts.

Participants demonstrated significantly higher IoF scores at the end of the assessment period (mean of last 3 attempts) compared to the start of the assessment period (mean of first 3 attempts) (t(9) = 9.471, p<0.001, d = 2.99). Although participant 7 demonstrated the same pattern of improvement as the other 9 participants, the participant was much slower in performing the tasks reaching a final IoF score approximately half that achieved by the other participants. **Fig 3** shows a box plot of the IoF scores; the outliers and/or lower bound of the whiskers represent participant 7’s data. SHAP performance appeared to plateau after 5–6 attempts as supported by a paired samples t-test, showing no significant difference between the IoF scores on the 6th and 10th attempts.

### Duration of contralateral hand involvement

Across the first and last 3 attempts, there were 1140 trials (19 tasks attempted, 6 attempts, 10 participants). 33 trials were excluded from the video analysis due to: (1) the task was incomplete when the 100s time limit elapsed (27 trials), (2) the camera did not have a clear view of the grasp/release being made (2 trials), and (3) the camera memory card was corrupt (4 trials).

Full results are presented in **Table 2**.

**Table 2:** Summary statistics relating to contralateral hand use.

	Grand Mean (SD)	Grand Median (IQR)	t-test	Cohens-d	Shapiro-Wilks test of normality	Skewness and Kurtosis
**Contralateral hand use during first 3 attempts vs the last 3 attempts**	6.47 ± 2.21s	2.06 ± 3.27s	t(9) = 4.040**	d = 1.28	W(10) = 0.963	Skew: z = -0.49
	4.68 ± 1.49s	2.29 ± 1.20s	p = 0.003		p = 0.819	Kurt: z = -0.71
**Grasping vs Releasing**	3.22 ± 1.31s	0 ± 2.14s	t(9) = 2.965*	d = 0.94	W(10) = 0.842*	Skew: z = 0.84
	2.33 ± 0.50s	1.50 ± 0.67s	p = 0.016		p = 0.047	Kurt: z = -1.13
**Grasping during first 3 attempts vs the last 3 attempts**	3.89 ± 1.54s	0 ± 2.47s	t(9) = 4.822**	d = 1.52	W(10) = 0.960	Skew: z = -0.50
	2.59 ± 1.19s	0 ± 1.53s	p = 0.001		p = 0.786	Kurt: z = -0.47
**Releasing during first 3 attempts vs the last 3 attempts**	2.58 ± 0.71s	1.56 ± 0.90s	t(9) = 2.336*	d = 0.74	W(10) = 0.974	Skew: z = -0.00
	2.10 ± 0.47s	1.49 ± 0.60s	p = 0.044		p = 0.926	Kurt: z = -0.64
**Direct vs Indirect interactions**	2.52 ± 1.10s	1.30 ± 1.25s	t(9) = -0.918	d = -0.29	W(10) = 0.952	Skew: z = -0.48
	3.03 ± 1.35s	0 ± 0s	p = 0.382		p = 0.698	Kurt: z = -0.33
**Direct interactions during first 3 attempts vs the last 3 attempts**	2.90 ± 1.54s	1.33 ± 1.32s	t(9) = 2.037	d = 0.64	W(10) = 0.932	Skew: z = 1.28
	2.16 ± 0.87s	1.25 ± 0.97s	p = 0.072		p = 0.469	Kurt: z = 0.65
**Indirect interactions during first 3 attempts vs the last 3 attempts**	3.56 ± 1.65s	0 ± 0s	t(9) = 3.666**	d = 1.16	W(10) = 0.846	Skew: z = 1.77
	2.53 ± 1.18s	0 ± 0s	p = 0.005		p = 0.052	Kurt: z = 0.47

**Correlation is significant at the 0.01 level,

*Correlation is significant at the 0.05 level.

For Kurtosis and Skewness |z|>1.96 is significant at p<0.05, and |z|>2.58 is significant at p<0.01 [24].

#### Overall hand use

The grand mean (±SD) time the contralateral hand was engaged in grasping and/or releasing reduced significantly from 6.47 ± 2.21s across the first 3 attempts to 4.68 ±1.49s across the last 3 attempts (t(9) = 4.040, p = 0.003, d = 1.28).

#### Grasp vs release

For 9/10 participants most of this contralateral hand involvement supported the initiation of a grasp rather than a release. The grand mean (±SD) time spent using the contralateral hand to assist with grasping (3.22 ±1.31s) was significantly longer (t(9) = 2.965, p = 0.016, d = 0.94) than during release (2.33 ±0.50s). Note that the assumption of normality was violated for this sample according to the Shapiro-Wilks test of normality, however, the z scores for skewness and kurtosis were not significant, suggesting a normal distribution.

**Fig 4:**
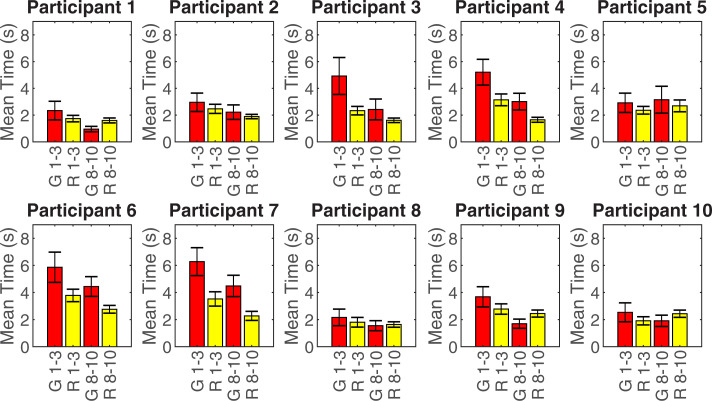
Mean duration of contralateral hand use during grasping/releasing for the first (1–3) and last (8–10) 3 attempts. G 1–3 = Involvement in grasp during attempts 1–3, R 1–3 = Involvement in release during attempts 1–3, G 8–10 = Involvement in grasp during attempts 8–10, R 8–10 = Involvement in release during attempts 8–10. Error bars show the standard error.

For both grasping and releasing, the time spent using the contralateral hand reduced significantly with practice. For grasping, the grand mean time the contralateral hand was involved reduced from 3.89 ±1.54s to 2.59 ±1.19s (t(9) = 4.822, p = 0.001, d = 1.52); whilst for releasing the grand mean reduced from 2.58 ±0.71s to 2.10 ±0.47s (t(9) = 2.336, p = 0.044, d = 0.74) (**Fig 4**).

#### Direct vs indirect

**Fig 5:**
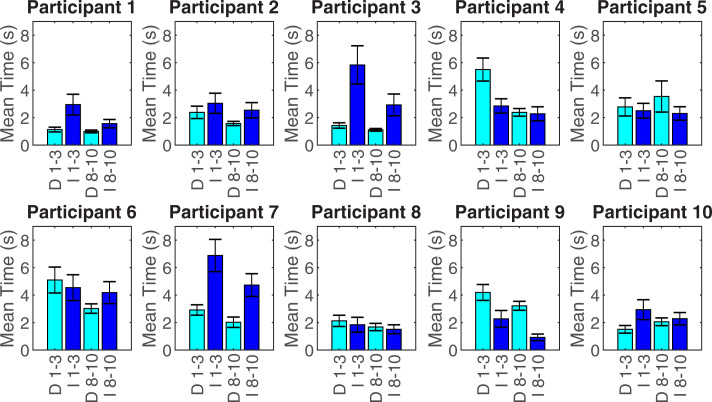
Mean duration of direct/indirect contralateral hand interactions for the first (1–3) and last (8–10) 3 attempts. D 1–3 = Direct interaction during attempts 1–3, I 1–3 = Indirect interaction during attempts 1–3, D 8–10 = Direct interaction during attempts 8–10, I 8–10 = Indirect interaction during attempts 8–10. Error bars show the standard error.

The use of direct and indirect contralateral hand interactions varied widely between participants (**Fig 5**). Consequently, the grand mean (±SD) time spent using the contralateral hand to directly contact the prosthesis (2.52 ±1.10s) was not significantly different (p = 0.382) to the time spent using the contralateral hand to indirectly operate the prosthesis mechanism by interacting with the object (3.03 ±1.35s). Nevertheless, within participant, the pattern tended to stay the same over learning.

The grand mean time spent using the contralateral hand to indirectly interact with the prosthesis, by using the object to operate the prosthesis mechanism, reduced significantly with practice from 3.56 ±1.65s to 2.53 ±1.18s (t(9) = 3.666, p = 0.005, d = 1.16). The grand mean time spent using the contralateral hand to directly interact with the prosthesis did not change significantly (p = 0.072) between the first 3 attempts (2.90 ±1.54s) and the last 3 attempts (2.16 ±0.87s).

### Additional observations

The Self-Grasping Hand was lightweight (<300g) and could be used with or without a cosmetic glove. When used without a glove, it was very difficult to grasp flat or rigid objects, due to the discrete positioning of the fingers (caused by the ratchet) and the rigid fingertips. Nevertheless, the hand mechanism was generally easy to operate, including finding the release switch. With the addition of a glove, the small amount of compliance in the fingertips and increased coefficient of friction improved the grasp of rigid objects; however, the working mechanism of the hand was slightly compromised, with reduced thumb mobility, higher strain on internal parts, and lower visibility of the release switch.

In common with other functional prostheses, donning the glove proved difficult. After testing with 3 participants using the PVC glove, an internal spring broke and the hand was returned to TU Delft and a decision was made to replace the hand. Exchanging the PVC glove for a silicone glove reduced the strain on the working mechanisms, but on some occasions, users found that the thumb would be pushed into a different position by the object being grasped. Further the silicone glove proved significantly less durable than the PVC glove. After the first data collection, holes began to appear at the thumb, and by the end of testing of the last participant, the thumb was sticking out of the end of the glove (see S3 File).

We observed that the relatively small aperture of the prosthesis (see S3 File), prevented the participants from being able to grasp for example, a standard sized jar (~70-75mm diameter). For the tasks people were able to complete, many were achieved using different grasps to those identified in the SHAP guidelines. For example, some participants grasped the cylinder from the top instead of using a power grip (it should also be noted that for all who used a power grip to grasp the cylinder, the thumb sat on top of the cylinder due to the narrow aperture of the hand, turning the grip into a hook grasp. Therefore, the thumb did not contribute to a strong power grip on the cylinder), other participants also slotted the key and the knife between their fingers rather than using a typical grasp. Full details are included in **Table 3**. Additionally, participants found it necessary to perform several compensatory movements, such as leaning the torso or lifting the elbow through abduction and internal rotation of the shoulder, likely in part caused by the absence of active wrist movement.

**Table 3:** Summary of grasps used for each task.

	Expected grasp	Tip	Lateral	Tripod	Spherical	Power	Extension	Other
**Task 1 Abstract Objects Light Sphere**	**Spherical**				59			
**Task 2 Abstract Objects Light Tripod**	**Tripod**	29	14	17				
**Task 3 Abstract Objects Light Power**	**Power**					51		9 approached the object from the top
**Task 4 Abstract Objects Light Lateral**	**Lateral**	2	38					11 approached the object from the top and 8 slotted the handle between their fingers
**Task 5 Abstract Objects Light Tip**	**Tip**	16	39	5				
**Task 6 Abstract Objects Light Extension**	**Extension**	1	5				54	
**Task 8 Abstract Objects Heavy Tripod**	**Tripod**	31	10	18				
**Task 9 Abstract Objects Heavy Power**	**Power**					42		16 approached the object from the top
**Task 10 Abstract Objects Heavy Lateral**	**Lateral**	5	34					12 approached the object from the top and 9 slotted the handle between their fingers
**Task 11 Abstract Objects Heavy Tip**	**Tip**	15	42	3				
**Task 12 Abstract Objects Heavy Extension**	**Extension**	1	18				41	
**Task 15 ADL Food Cutting**	**Power**		16	3				12 slotted the knife between their fingers, 12 combined a power grip with slotting it through their fingers, and 5 reversed the orientation of the knife and used a combination of a power and lateral grip to stabilise the handle
**Task 18 ADL Glass Jug Pouring**	**Lateral**		32			28		
**Task 19 ADL Carton Pouring**	**Power**					60		
**Task 22 ADL Lifting a Tray**	**Lateral or Extension**		34				26	
**Task 23 ADL Rotate Key**	**Lateral**	1	28					30 slotted the key between their fingers
**Task 24 ADL Open / Close Zip**	**Lateral or Tip**	7	30					10 pushed the zip with their fingers
**Task 25 ADL Rotate a Screw**	**Power**	2	6			50	1	
**Task 26 ADL Door Handle**	**Power**					42		17 opened the door handle with an open hand either hooking their fingers or using the side of their index finger

Some participants used grasps different to those identified in the SHAP guidelines, the suggested grasp is detailed under ‘Expected Grasp’, followed by the number of participants who approached the task with each type of grasp for each attempt (note that only successful attempts are included in these figures). For some tasks, tip, lateral, and tripod grips were used almost interchangeably. This usually related to the exact thumb position chosen.

## Discussion

Consistent with our first hypothesis we observed a higher mean SHAP IoF score in the last three attempts compared to the first three. Similar rapid improvements in IoF scores have been demonstrated during repeated testing of anatomically intact participants on first introduction to myoelectric [10, 25] or body-powered [26] prosthesis simulators. This suggests that the process of learning to use the Self-Grasping Hand may be similar to the learning processes for other types of prosthesis. The mean IoF scores presented in this study (mean = 17–33) are slightly lower than those presented in studies using body-powered and myoelectric prostheses and prosthesis simulators (~30–70). This may be accounted for by the small aperture of the Self-Grasping Hand. Kyberd et al. [13], reported a mean IoF score of 28 ±14.2 for small aperture hands. It is interesting to note that although participant 7 achieved a lower baseline IoF than the other participants, they also showed a clear improvement in their IoF, and the time the contralateral hand was used to assist with grasp and/or release was similar to other participants.

Consistent with our second hypothesis, we also observed a significant reduction in the amount of time the contralateral hand was involved in the operation the Self-Grasping Hand. However, it is worth noting that although the mean time spent using the contralateral hand reduced by around 1.8 seconds between the first and last 3 attempts at SHAP, the contralateral hand use normalised to task duration showed a small increase (from 29% to 32%). This may be a ‘floor’ effect–for example, it may in practice be very difficult to involve the contralateral hand for less than a second or so.

The contralateral hand was occupied longer during grasping activities than releasing, which is perhaps surprising, given the involvement of the contralateral hand is almost always required for release. This may be explained by the fast action of the releasing mechanism, compared to the more precise hand placement required to form a secure grasp. It was also interesting to note that the participant-specific pattern of direct/indirect use of the contralateral hand, appeared relatively stable over practice. If future work was to identify one particular pattern of use to be more beneficial, then training to encourage this type of use may be worth exploring. As noted in the introduction, this methodology was focussed on exploring the use of the contralateral hand; therefore, this study did not investigate changes in the use of direct grasps, where the hand would grasp an object without the assistance of the contralateral hand. In future it would be interesting to understand whether the reductions in contralateral hand use coincided with more direct grasping with the prosthesis, or whether the type of grasp (direct or indirect) remained the same, but the speed of the contralateral hand interactions increased. It would also be interesting to use our methods to explore the nature of contralateral hand use during real-world use of both active and passive prostheses. The work of Spiers et al. [27] created a taxonomy covering both prosthetic and contralateral hand involvement during ‘at-home’ use of three different (active) prostheses, but did not examine changes to contralateral hand use with practice. Real world evaluations of the Self-Grasping Hand would give greater insight into the value the user places on the device, and whether the extent of contralateral hand involvement proves to be a major problem for users.

Of the 1107 trials included in the video data analysis, participants exceeded the SHAP time limits (8*mean of the normative data) in 296 trials (27%), and in 52 of these (5%) the time limit was exceeded by the contralateral hand involvement alone, before considering the time taken to perform the rest of the task. The myoelectric prosthesis users in a study by Burgerhof et al. [12] demonstrated similar difficulties completing the tasks within the specified time limits, particularly struggling with the pick-up coins, rotate a screw, and food cutting tasks. Our participants were not required to undertake the pick-up coins task, however, 79% of the food cutting trials and 70% of the zip task trials timed out. All other tasks were completed within the time limits over 50% of the time. However, only the tray task, which is bilateral was completed by all participants within the time limit every time. To address this, we would recommend a re-design of the Self-Grasping Hand to increase the overall available aperture and the number of discreet aperture settings, or increase the compliancy of the fingers or the mechanism. Additionally, improvements are needed to the design of the Self-Grasping Hand to ensure it is sufficiently robust, and consideration of the type of glove to be used with the hand is needed.

One limitation of the current study is that we were unable to assess the contralateral hand involvement for an experienced prosthesis user. A person with experience using the limited degrees of freedom available with a typical prosthetic hand, would be better able to adapt to the constraints of grasping using a prosthetic hand and the way it conforms around and reacts with objects. Although it is also worth noting that a person with no experience of prosthesis use, may adapt differently (and potentially faster) to the novel mechanism than a person who is used to using a prosthetic hand which operates in a specific manner.

## Conclusion

This study involved the development of a set of guidelines to assess contralateral hand involvement in grasping and releasing using a prosthesis. It also involved assessment of the contralateral hand involvement when using a passive adjustable prosthesis, the Delft Self-Grasping Hand. The Self-Grasping Hand is a novel and promising prosthesis. Participants in our study showed improvements in functionality score over the first few attempts at the Southampton Hand Assessment Procedure (SHAP). As hypothesised, the involvement of the contralateral hand showed a significant reduction from 6.47 to 4.68 seconds (t(9) = 4.040, p = 0.003, d = 1.28). However, contrary to our hypothesis, the contralateral hand was engaged for significantly more time in grasping than releasing (t(9) = 2.965, p = 0.016, d = 0.94). Our evaluation suggests that the hand is more functional than a static passive hand with no grasping function. However, the aperture of the current design is too small for some of the SHAP tasks. Further, when using ‘off-the-shelf’ cosmetic gloves with the Self-Grasping Hand, these either reduce the performance of the hand (PVC), or are highly prone to damage (silicone). With some improvements to the aperture size and the robustness of the hand, the Self-Grasping Hand could offer a functional, lightweight and good-looking alternative solution.

## Supporting information

S1 File Example grasp/release video. (MPEG)

S2 File Summary data. (XLSX)

S3 File Self-Grasping Hand dimensions. (DOCX)

S1 Raw image (JPG)
