# Supplementary material for: An evaluation of contralateral hand involvement in the operation of the Delft Self-Grasping Hand, an adjustable passive prosthesis
Source: PLoS One. 2021 Jun 17;16(6):e0252870. doi: 10.1371/journal.pone.0252870 (PMC8211290; doi:10.1371/journal.pone.0252870)
Supplement: S3 File — (DOCX) [file pone.0252870.s003.docx]

Supplementary material to: An evaluation of contralateral hand involvement in the operation of the Delft Self-Grasping Hand, an adjustable passive prosthesis

**Self-grasping hand dimensions**

All measurements were taken whilst wearing a silicone glove which prevented the thumb from remaining in a fully abducted position (the elasticity of the glove reduced the abduction by 10-15°). **Thumb abducted – Hand fully open**

A: Tip of middle finger to base of hand = 14cm

B: Tip of thumb to tip of index finger = 4cm

C: Tip of thumb to tip of little finger = 7cm

D: Base of thumb (excluding web) to base of little finger = 7cm

E: Width of palm = 7cm


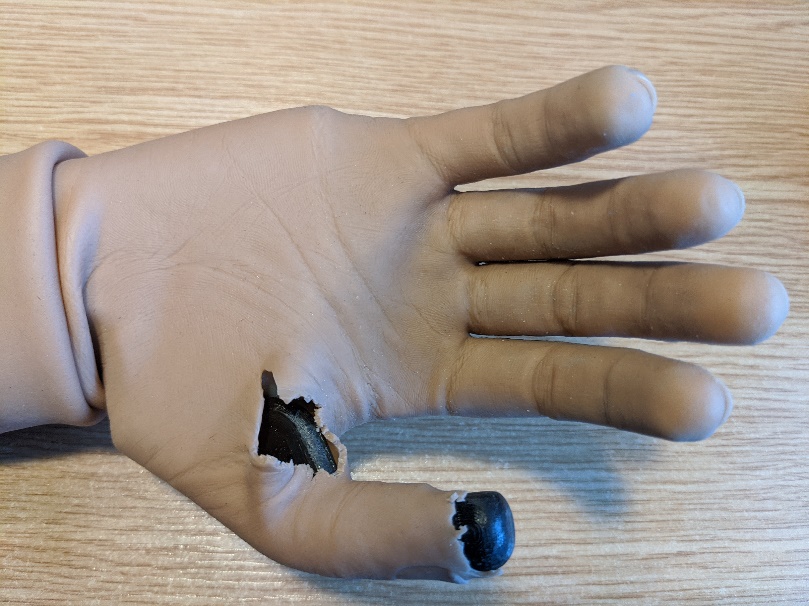


A

B

C

D

E

**Thumb abducted – Hand fully closed**

F: Tip of middle finger to top of palm = 5.5cm

G: Tip of middle finger to base of palm = 9cm


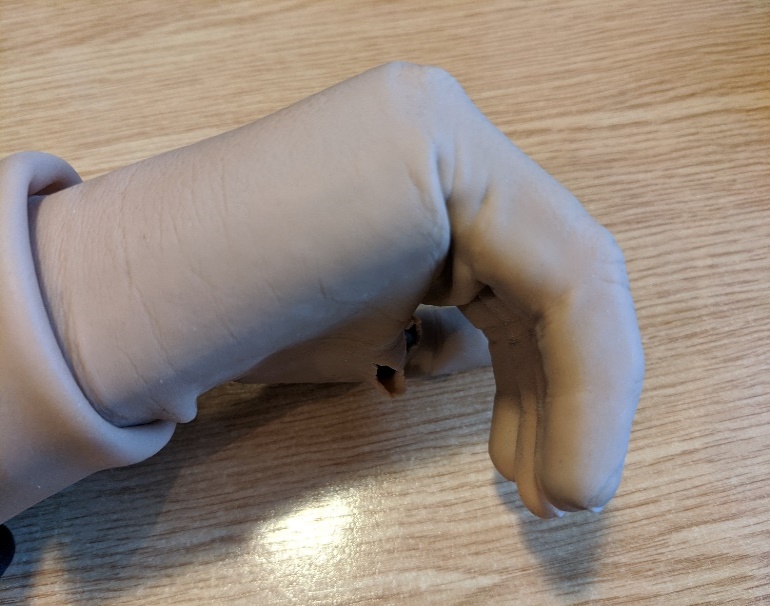


F

G

**Thumb in opposition – Hand fully open**

H: Tip of index finger to tip of thumb = 3.5cm

I: Aperture of opening between index finger and thumb = 3.5cm


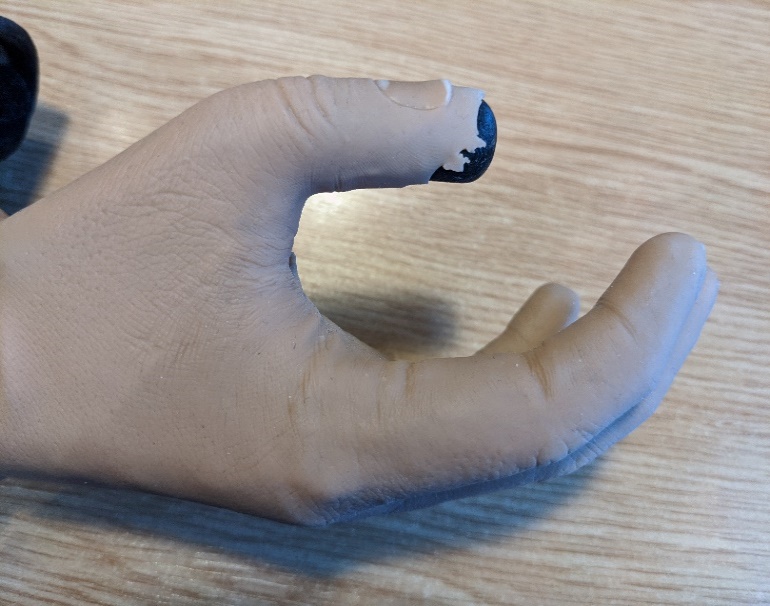


H

I

**Thumb in opposition – Hand partially closed (pinch grip)**

J: Tip of middle finger/thumb to web of glove = 5cm

K: Aperture of opening between index finger and thumb = 3cm


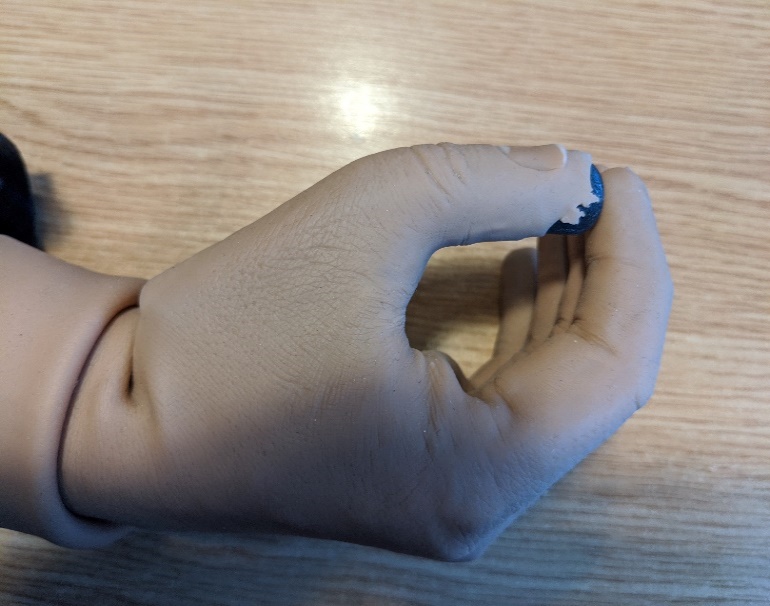


K

J
