# Supplementary figures and images for: An evaluation of contralateral hand involvement in the operation of the Delft Self-Grasping Hand, an adjustable passive prosthesis
Source: PLoS One. 2021 Jun 17;16(6):e0252870. doi: 10.1371/journal.pone.0252870 (PMC8211290; doi:10.1371/journal.pone.0252870)

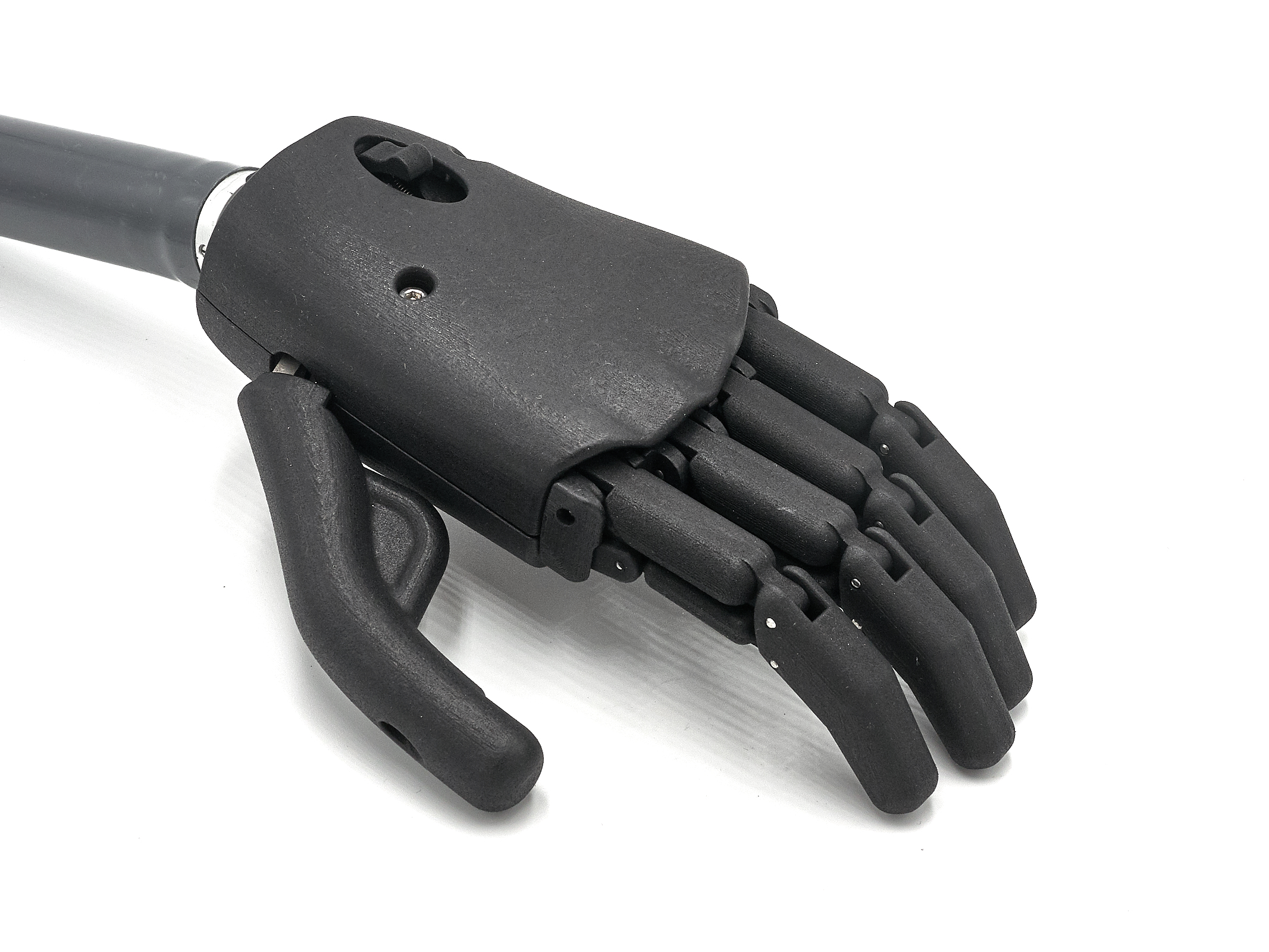

Supplement: S1 Raw image — (JPG) [file pone.0252870.s004.jpg]
